# Identifying technology innovations for marginalized smallholders-A conceptual approach

**DOI:** 10.1016/j.techsoc.2017.03.002

**Published:** 2017-05

**Authors:** Mohammad Abdul Malek, Franz W. Gatzweiler, Joachim Von Braun

**Affiliations:** a University of Bonn-Center for Development Research (ZEF), BRAC Research and Evaluation Division (RED), Walter-Flex-Str. 3, 53113 Bonn, Germany; b University of Bonn-ZEF, Chinese Academy of Sciences -Institute of Urban Environment, Walter-Flex-Str. 3, 53113 Bonn, Germany; c University of Bonn-ZEF, Walter-Flex-Str. 3, 53113 Bonn, Germany

**Keywords:** Ex-ante approach, Marginality hotspots, Technological and institutional innovations, Green revolution, Small holders' potentials, Business plan

## Abstract

This paper adds a contribution in the existing literature in terms of theoretical and conceptual background for the identification of idle potentials of marginal rural areas and people by means of technological and institutional innovations. The approach follows ex-ante assessment for identifying suitable technology and institutional innovations for marginalized smallholders in marginal areas-divided into three main parts (mapping, surveying and evaluating) and several steps. Finally, it contributes to the inclusion of marginalized smallholders by an improved way of understanding the interactions between technology needs, farming systems, ecological resources and poverty characteristics in the different segments of the poor, and to link these insights with productivity enhancing technologies.

## Highlights

• An ex-ante approach for identification of idle potentials of marginal rural areas and small holders by means of agricultural technology and institutional innovations.
• The approach is divided into three main parts (mapping, surveying and evaluating) and several steps.
• It guides actions to overcome current barriers to technology access and adoption for improving agricultural productivity.

## 1 Introduction

The first Green Revolution (GR1) was just one aspect of a much larger transformation of global agriculture in the developing world during the 20th century [22]. The success of surprising crop productivity growth was caused mainly by the technological development of crops through the combination of high rates of investment in crop research, infrastructure, and market development and appropriate policy support [39]. Although GR1 impacted positively to productivity improvement, fall in real food prices, poverty reduction and food security, it was not always the right answer for solving the numerous problems of poverty, food security, and nutrition facing the poor. GR1 was very often criticized for its uneven social and spatial distribution effects.^1^ Benefits have been widespread only in favorable areas but not in unfavorable marginal and less favored areas^2^ (LFAs)in Africa and Asia (hereinafter we will use only the term “marginal areas”), the potential impacts on economic growth, poverty and self-sustaining development have not yet been brought out [12], [13], [28], [39]. In last decade, in the context of rising food prices and growing population, the global community including donors, governments, philanthropists have begun to refocus attention on agriculture [6]. Thus, it is assumed that the marginal areas continue to rely on agricultural productivity as an engine of growth and hunger reduction [52], [24].

The traditional ‘pipeline’ approach, in which researchers develop new technologies and pass them to extension agents who in turn are meant to persuade farmers to adopt them, was abandoned in favor of a more inclusive and holistic approach applying to individuals and institutions at all levels. Because of the passive role of the end-users the pipeline approach for agricultural technology innovations has produced less than satisfactory returns on considerable investment for sub-Saharan Africa [25]. In response to those insights, the international development partners, for example, the International Fund for Agricultural Development (IFAD) and the World Bank are following the innovation systems approach that has no formal innovation pipeline or standard criteria for selecting or identifying innovations [40], [41]. In such approaches, the poor small holders (SHs) are not only as integral part of the innovation system but as valuable source of the innovation process [19]. Some other innovative thinking relating to business solution and the use of ICT in agriculture have been pursued for the last decades. Jugannd or frugal innovation [42], social innovation and entrepreneurship for the poor, rural communities and business at bottom of the pyramid (BOP) with appropriate marketing practices are promising examples.

However, the development approach is not necessarily being holistic or sustainable. The need for continued investments in agricultural innovation and productivity growth is as important today as it was in the early years of the GR1. Unfortunately, investment in agriculture dropped off dramatically into the mid-2000s [23] in [39]. Since the mid-2000s and heightened after the 2008 food price hikes, there has been continued interest in agricultural investment, and there are repeated calls for GR2 type activities [5], [9]. Building on the lessons learnt from the GR1, international development partners, for example, AGRA aims at a strategy to transform today's rural poverty into tomorrow's prosperity by sustainably and significantly increasing the productivity of SHs [4], [48].

Despite progress in agricultural productivity and poverty reduction, still some 40% of rural population of developing countries are estimated to live in marginalized conditions [26], [27], [38]. After the GR1, it was soon realized that “one size does not fit all” did not benefit the marginalized poor. A better targeted approach was required to exploit the potentials of particular segments of poor households and communities [46], [47] in their particular ecological and institutional environments. For that reason, [49], for example, advocated for government strategies to be tailored to different strata of farmers at hinterland zones. To that date, however, a comprehensive assessment approach was lacking. The marginality perspective [50] helped to refocus attention on the nexus of poverty, exclusion and ecology and thereby better recognize the systemic links between agro-ecological potentials and human capabilities which can be triggered for productivity growth by technological and institutional adjustments. Thus, there are three main innovative aspects to the ex-ante analysis we propose here, which to the best of our knowledge, are not addressed in any other ex-ante assessment for productivity growth in agriculture:

1. The combination of ecological, technological and institutional dimensions in the assessment,
2. The inclusion of marginalized SHs and marginalized land areas, and
3. The targeted approach towards different segments of the marginalized poor.

In our approach, identifying marginalized land areas which could be brought into agricultural production is a straightforward objective of ex-ante assessments which aim at agricultural productivity growth. Suitable land for growing crops is obviously a critical production factor. Identifying those areas is however of little value to the aim of increasing productivity and income of marginalized SHs, if they do not have access to the land and are not provided an enabling institutional and technological environment to benefit from cultivating the land. In fact, those ecological, technological and institutional constraints prevent the marginalized poor from developing their capabilities.

The ex-ante assessment we propose here is not something to be discovered through evaluative research rather it creates a thorough understanding of the interactions between technology needs, farming systems, ecological resources, institutional and poverty characteristics in the different segments of the poor. The insights can be used to guide action to overcome current barriers to technology access and adoption for the policy makers and practitioners working for improvement in productivity growth of the marginalized SHs in marginal areas. A manual has been published which describes the detailed step-by-step approach of the assessment [29] and examples of applying core elements of the ex-ante assessment from India, Ghana and Bangladesh are presented in [14], [17], [33] and [31].

The next section reviews the theory of change and the common approach of the assessment. Section 3 elaborates on each of the steps of the assessment and the final section summarizes the approach and concludes.

## 2 Conceptual framework and theory of change

**Fig. 1:**
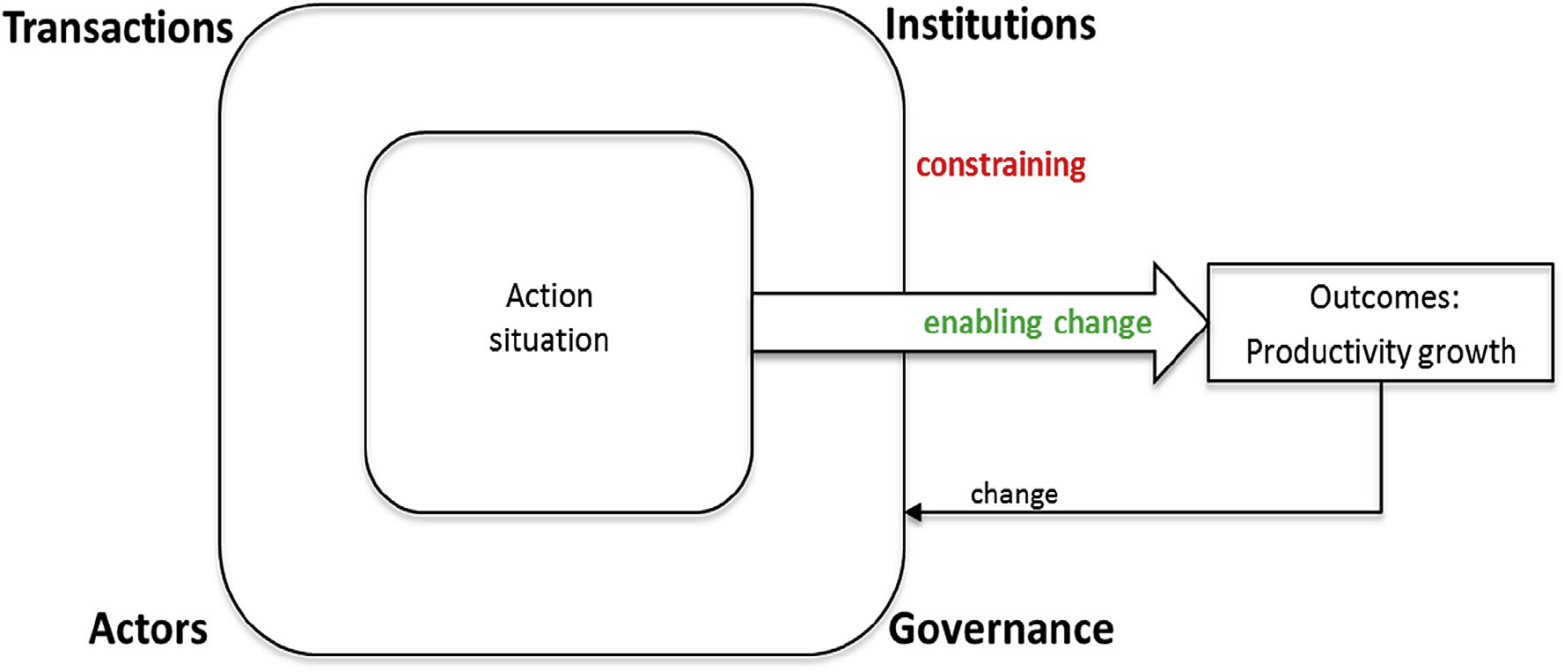
Conceptual framework and theory of change.

Source: Adjusted from [21] and [36]

In our framework, the poor (actors) are in action situations which are characterized by 1) particular types of transactions, 2) the actor characteristics and assets, 3) institutions which formally or informally rule behavior and define use and access to resources, especially property rights, and 4) governance structures. The configuration and effects of these four factors determine whether they work as barriers to innovation towards productivity growth, or as enablers. All four factors can be used as drivers of change so that they function less as inhibitors and more as enablers for technological or institutional innovations.

For explaining barriers to change which prevent the unleashing of the potentials of the poor [34] refers to limited access (in contrast to open access) orders, [1] and [2] refer to extractive (in contrast to inclusive) institutions, and [21] refers to segregative (in contrast to integrative) institutions. Despite the different use of terms all theories contribute to explaining that the poor are locked in action situations defined by institutions^3^ and governance structures which define the types of transactions the poor are engaged in and the conditions under which they live. From that perspective, it becomes obvious that poverty and marginality is to a large extent man-made. The institutions of marginality keep people marginalized and prevent them from making full use of their capabilities.

Both, physical and social dimensions of transactions are particularly relevant for the action situations the poor find themselves in: institutions and governance structures manifest existing types of transactions which do not set incentives for creative change, innovation, or competition. They make it too costly for the poor to change established types of behaviors. Although efforts to change towards productivity growth (e.g. by investing and saving) also require capital inputs [10], [44] argue that the poor have assets, but because of the prevailing institutions and governance structures, this, particularly land, is prevented from being used as capital, e.g. as collateral. Thereby the poor cannot make use of their “dead capital”.

[34] emphasizes the constraints that institutions and governance structures have on access to e.g. decision making in political markets, education and income opportunities, opportunities for progressing along the value chain, or access to transport, communication and information infrastructure. When institutions and governance structures are inclusive [1] and [2] or integrative [21], they change the ruling framing conditions in a manner that enable the poor to change manifested types of transactions. They create incentives to innovate, opportunities for alternative income sources, and do not prevent access to political and economic markets.

## 3 Steps of the ex-ante assessment of technology innovations for marginalized smallholders

**Fig. 2:**
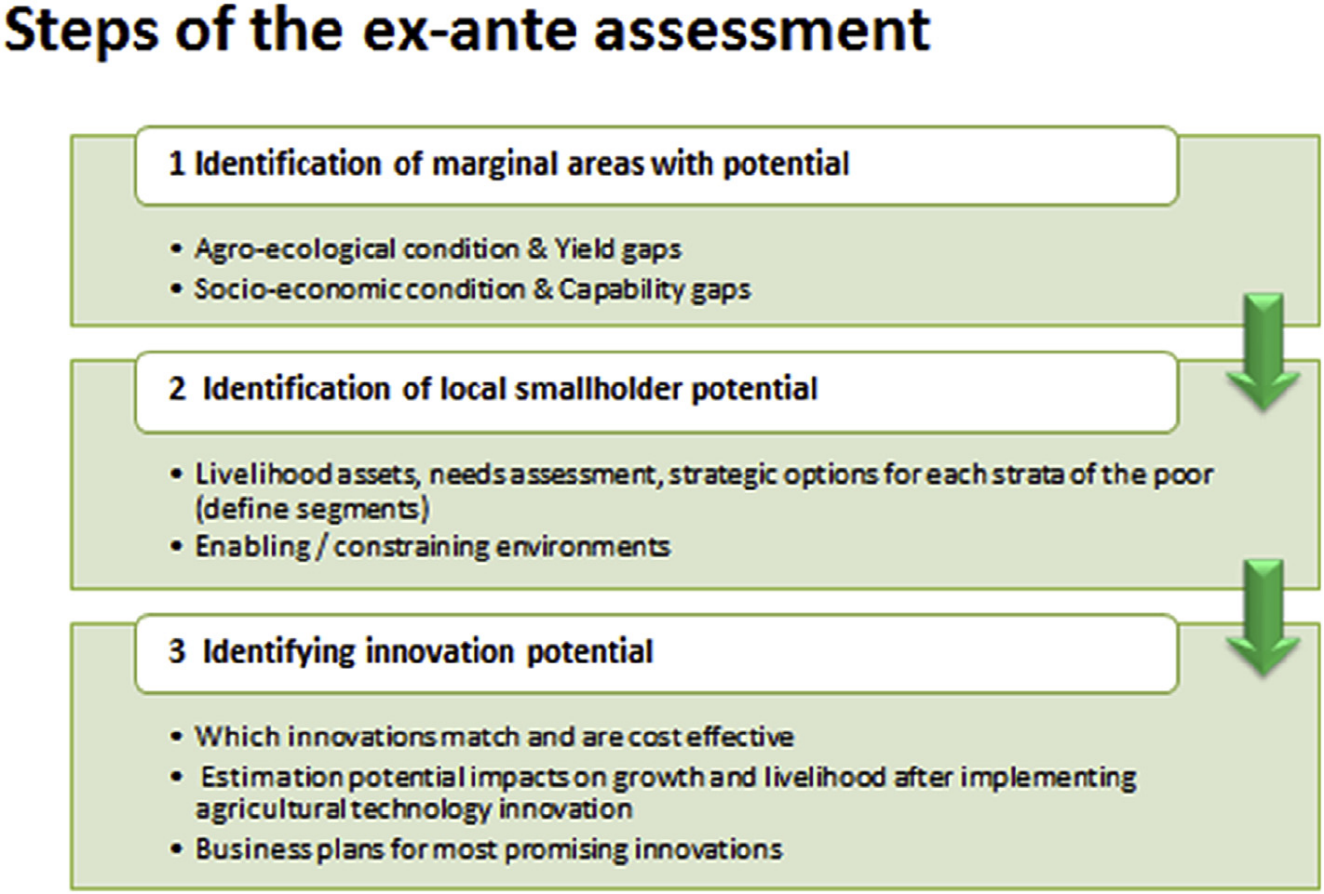
Steps of the TIGA ex-ante assessment.

### 3.1 Identifying marginality hotspots

This sub-section represents an approach to mapping marginality and identifying marginality hotspots with agro-ecological potentials ideally followed in South Asia and Sub-Saharan Africa. The approach supports the identification of people who live in socio-economically marginalized conditions and are located in agro-ecologically marginalized areas. The overlap of marginalized people and areas leads to the identification of unused peoples' capabilities and agro-ecological potentials. Areas in which both overlap are called “marginality hotspots” and could become the priority areas for development investment.

Poverty and marginality are two terms often used concurrently. Poverty measurements often inform about peoples' economic characteristics. Well-known is the assessment of poverty by indicating a person living below “one dollar a day” as poor which was introduced in the World Development Report (WDR) on Poverty in 1990 by the World Bank [43], [51] revisited that measure about 20 years later stating that an international comparison still needs to include country-specific information. Therefore, national poverty lines were used to come up with an adjusted measurement of poverty. Later the average poverty line was set at $1.25 [43]. Among the poor, adjustments were also made. For example, people living on $0.75 to $1 a day were defined as subjacent poor, those living on $0.50 to $0.75 a day, as medial poor and people living below 50 cents a day as ultra-poor [3]. A first approach to mapping global marginality hotspots was taken by [18] by making use of proxies representing spheres of life. Five so-called *marginality dimensions* were used to visualize global marginality hotspots. By defining cut-off points for all marginality indicators, the degree of marginality of countries in Sub-Saharan Africa and South Asia could be compared. Overlaying the different dimensions of marginality then helped to identify areas in which several dimensions of marginality overlap.

#### 3.1.1 Dimensions of marginality

**Fig. 3:**
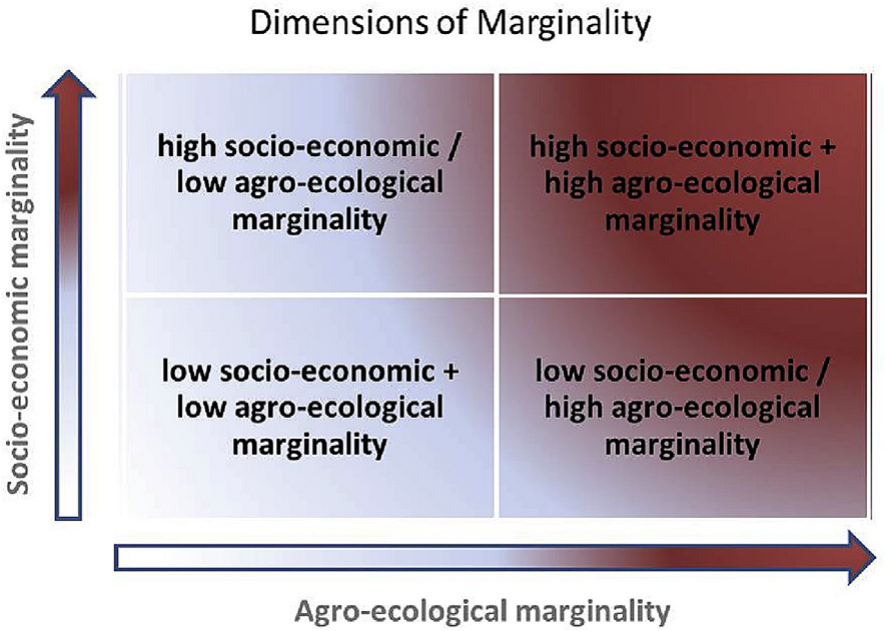
Concept of marginality mapping, Source: [17].

Both dimensions are mapped by using so-called conditional and positional indicators. The concept of marginality defines marginality not only by the assets a household owns but also by its access to infrastructure, public services and resources [16]. Accordingly, a poor household located adjacent to a river, a forest or a road, will be in a better position than one with the same endowments but which is located more remote. *Conditional indicators* therefore present the current state of an individual or household and its endowments, e.g. educational or income level, land ownership and other assets. *Positional indicators* refer to positions in physical and social space and indicate the potential to enhance the current condition of an individual or group in an identified area. Conditional indicators are e.g., access to education, access to markets, communication and transport infrastructure, or positions in social organizations or ethnically defines strata, which define rights to make decisions. Positional indicators within the agro-ecological dimension focus on the economic and technological situation of farmers, like access to finance, knowledge and technology.

#### 3.1.2 Capability and potential gaps

**Fig. 4:**
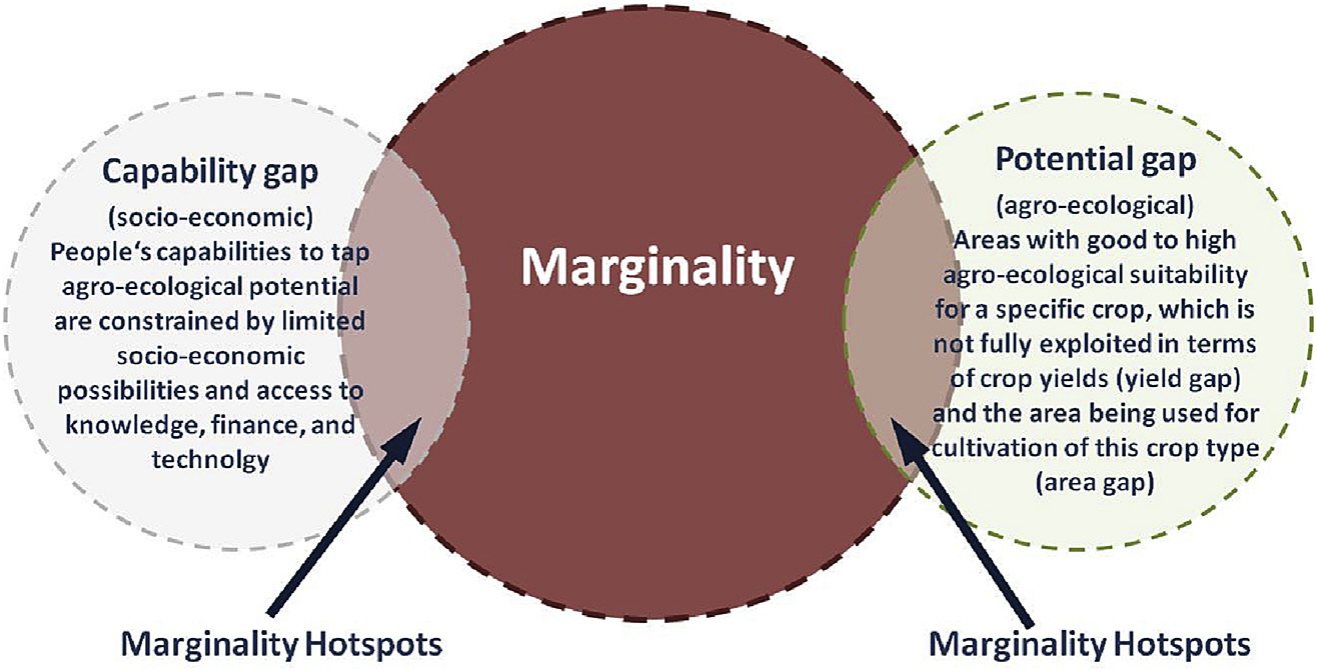
Mapping approach to define marginality hotspots.

Source: [17]

*Capability gaps* refer to the lack of socio-economic capability to use the agro-ecological potential. Here they are defined as unused socio-economic possibilities due to limited access to knowledge, finance, and technology. To identify capability gaps, we overlap information on agro-ecological suitability with data on positional agro-ecological marginality (farmers' access to credit, fertilizer, and advisory service) and compare the degree of marginality of both maps.

*Potential gaps* are defined as areas with good to high agro-ecological suitability for a specific crop, which are not fully exploited in terms of crop yields (yield gap) and the area being used for crop cultivation of this crop type (area gap). In order to identify potential gaps, we overlap areas with good to high agro-ecological suitability for Sorghum, Wheat and Maize – crops that are important to agriculture in any particular country/geography setting – with yield gaps and area gaps.

Hence, a capability gap is related to people and their capabilities, while the potential gap is related to land and its environmental characteristics such as climate, soil and topography. The overlap between marginalized areas (or people) and capability/potential gaps are defined as marginality hotspots, which are prospective for productivity growth and poverty reduction. Empirical examples can be found at [17] and [31].

### 3.2 Identification of smallholders' potentials

Once the marginality hotspots (e.g., sub-districts/district/state) are identified study villages within the marginality hotspots are selected. For this purpose, sub-district level statistics help to identify the villages. An initial visit by the research team at the locality and consultation at sub-district level with agricultural officers/field workers working in the selected sub-districts may be useful. Finally, a list of all marginal villages is prepared and villages are randomly selected for the assessment.

Next, a household census containing basic information mainly related with assets may be conducted for the entire households in the selected villages. Poor SH households are identified. For the categorization of poor (with different strata) and non-poor, principal component analysis (PCA) may be used. The categorization may be validated by participatory wealth ranking (PWR) exercise. By means of stratified random sampling, a selection from the different strata of poor SHs are made for the assessment. This may be the first level of stratification done only for the sampling purpose-final stratification is made based on the income criteria after the quantitative sample survey is conducted – an example can be found at [32]. The household self-perception about stratification is also collected during quantitative sample survey. However, the procedure for selecting the study villages and drawing samples varies from country to country.

The sustainable livelihoods framework (SLF) developed by DFID [7] can be used to improve understanding of livelihoods of the selected poor SHs. The livelihoods approach places households and their members at the center of analyses and decision making, with the implication that the household-centered methods of analyses must play a central role in developing an understanding of livelihood strategies. Applying SLF highlights the multilayered interactions between technologies and the vulnerability context of households – their asset base, access to social capital, and livelihood strategies. However, additional aspects of culture, power, and history are integrated to understand the role of agricultural research in the lives of the poor [8], [11].

The livelihoods analyses do not have to be exhaustive to be useful for determining the potentials of the poor SHs that can be developed for appropriate technology innovations. Rather than trying to develop a full understanding of all dimensions of the vulnerability context, the aim is to identify those capital assets, trends, shocks and aspects of seasonality that are of particular importance to livelihoods of the poor SHs. Effort can then be concentrated on understanding the impact of these factors and how negative aspects can be minimized.

In addition, need assessment can identify demands, wants and requirements for improving the quality of current livelihoods. Such needs can be discrepancies between current and needed or desired conditions of SHs and they are assessed to ensure that technological innovations which are economically possible also match the wants and aspirations of the poor – an important aspect which is also captured by allocating the surveyed SHs to the strategic options.

**Table 1:** Typical Data Requirements and Collection for the livelihoods assessment.

Livelihood component	Data requirements		Data collection tool (Qualitative)	Data collection tool (Quantitative)
	Indicator for discussion and analysis	Vulnerability context		
Human Capital	Labor, Education, Health, etc.	Disease epidemics (malaria, cholera, dysentery) due to poor sanitary conditions	In-depth Interview	House hold Survey
Financial Capital	Remittance, Deposit, etc.	Increased theft, Unemployment, tax	Wealth Ranking, Village workshops,	Household Survey, Community-level formal surveys
Natural Capital	Land, Irrigation water etc.	Drought, Flooding, Land degradation, Pests	Social mapping, participatory resource mapping, transect walks	Household Survey, Community-level formal surveys
Physical Capital	Machinery, Tools, Livestock	Stricter loan requirements, Price shocks, Rapid inflation	Wealth ranking, Village workshops	Household Survey, Community-level formal surveys
Social Capital	Claims, kinship networks, safety-nets etc.	Recurring environmental shocks, Breakdown ability to reciprocate, Morbidity and Mortality affect social capital	In-depth interview, Key Informant interviews	Household Survey, Community-level formal surveys

**Fig. 5:**
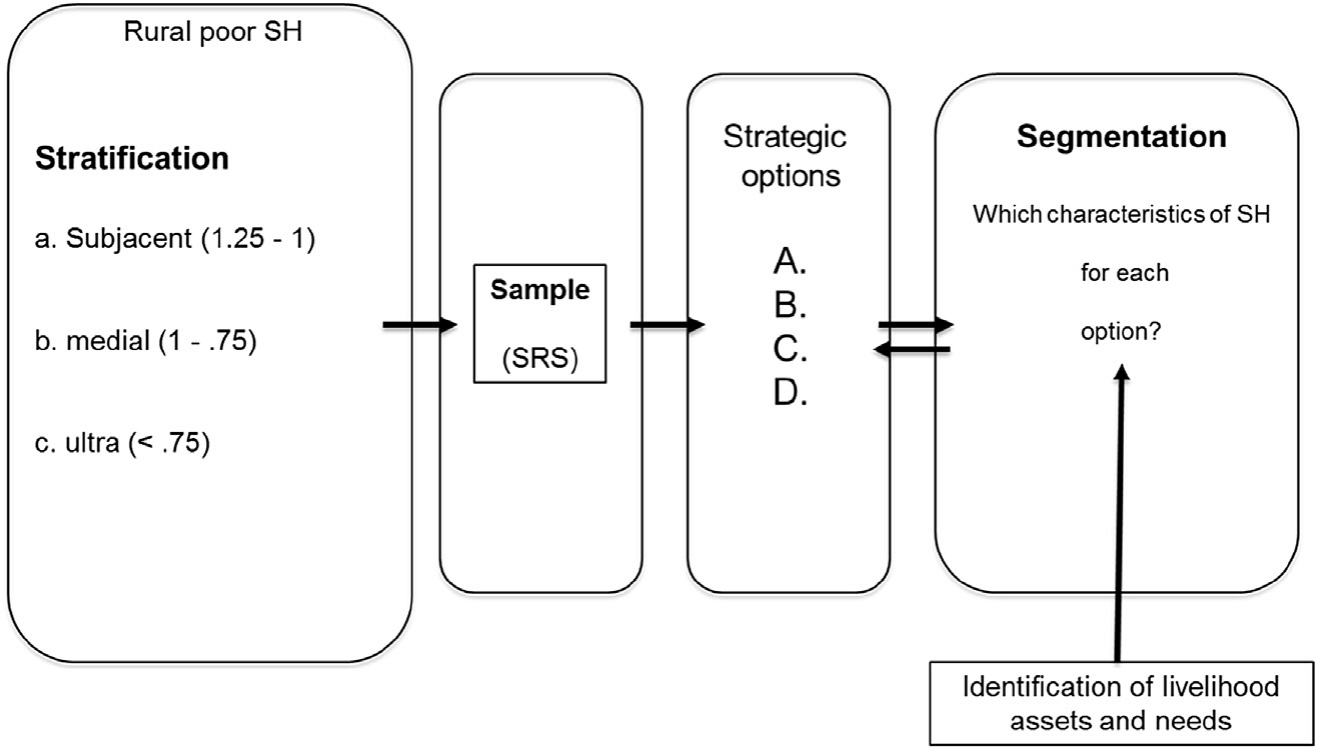
From stratification to segmentation.

This grouping of poor households from different strata is carried out in parallel with the livelihood and need assessment done in the previous step. This can be done in a participatory manner and supported by agronomic calculations based on household data from the livelihood assets and needs assessment to ensure that the options are realistic (no wish lists) and economically viable for each of the actors from different strata.

The segments are defined for each strategic option. Segmentation is necessary to identify suitable innovations – innovations which match the characteristics of the households in each segment and thereby contribute to achieving the overall goal of increasing productivity. For example, all SHs allocated to option A own land, lease land or are sharecroppers and each belong to a different income category. Land and income define different segments which can be defined by additional characteristics, such as family members, level of education and social status. After this step in the assessment we know which strategic options are available for which strata of the poor and which characteristics the poor have in each option category (segment). Finally, the quantitative sample survey data may also be analyzed using cluster analysis to evaluate different strategic options. Cluster analysis may be performed using a sequence of a common hierarchal and exchange algorithm. Then the identified strategic options may be validated by demonstrating the correlation between them and independently reported options.

Step 4: Identifying proximate and underlying barriers to technology adoption.

**Fig. 6:**
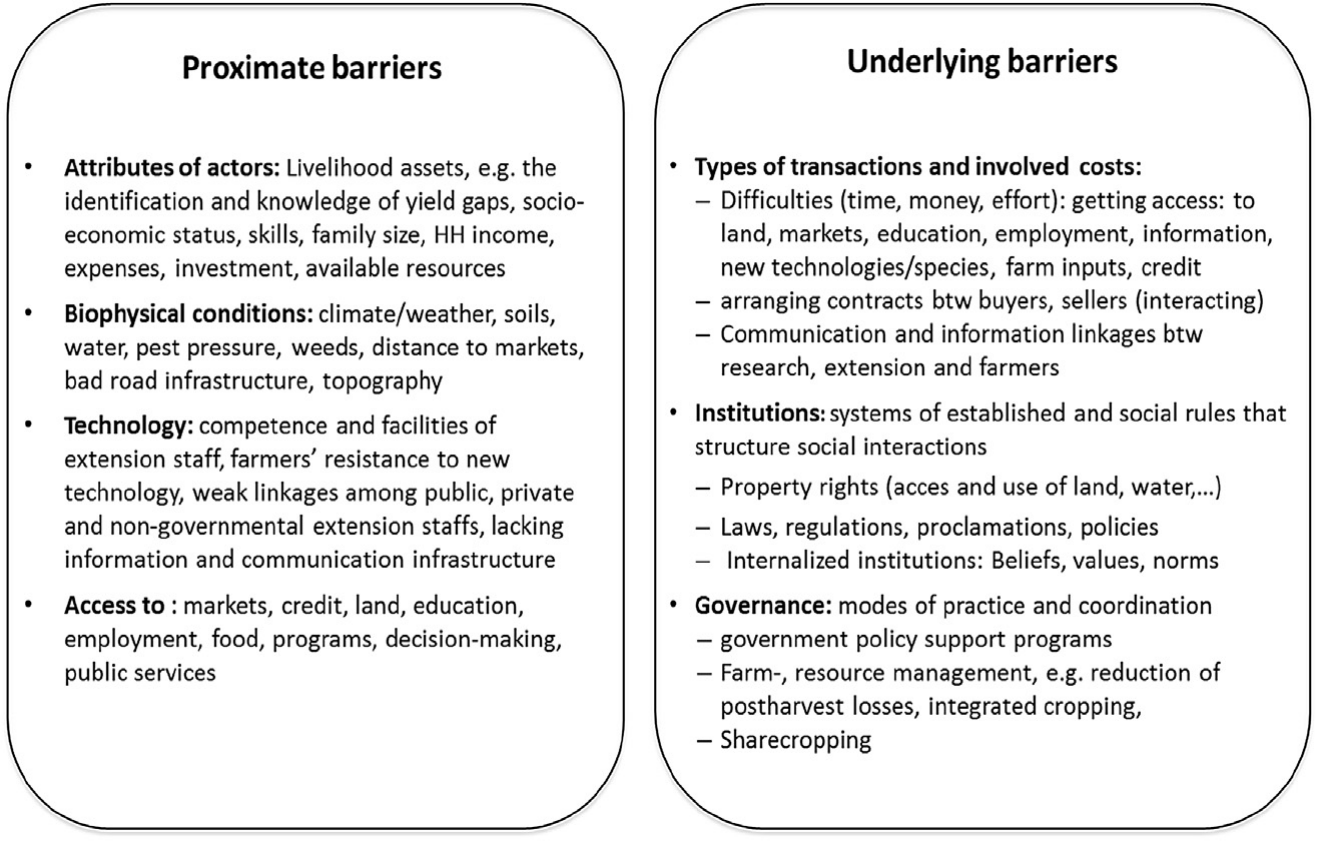
Proximate and underlying barriers.

### 3.3 Identifying and estimating innovation potentials and packages

Instead of the traditional top-down ‘pipeline’ approach this paper proposes a bottom-up approach by matching available agricultural technologies with the circumstances in which the poor live. For that reason, no particular productivity enhancing technology is being pre-selected or promoted. Whether agricultural technology innovations are what the rural poor want and which one suit which segment of the poor will be identified during the process of the ex-ante assessment.

Bundles or packages of innovations, integrated innovation measures or systems include the innovation itself and the enabling environment. Enabling environments refer to the livelihood dimensions and can refer to the legal environment and institutional infrastructure (e.g. property rights) or knowledge required to make use of the innovation. Communication and transport infrastructures can also be necessary enabling environments. The starting point of identifying potential productivity enhancing innovation packages should be with current farming/management practices and technologies. Innovations can also include institutions or policies and new products, production processes, cheaper inputs, improved distribution and marketing and even improved ways of innovating. Innovation can stress the value of linking ‘old and new’ or traditional knowledge and practice and new, externally introduced ones.

**Fig. 7:**
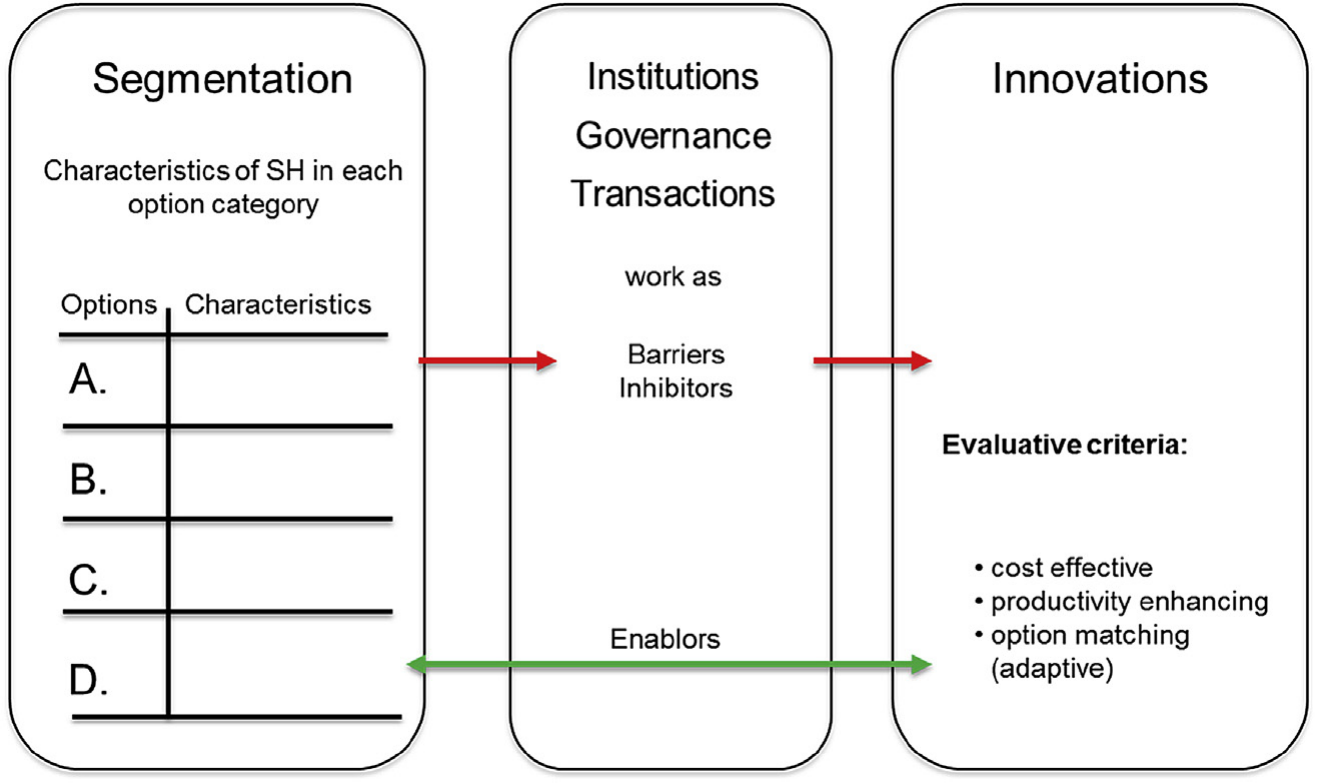
Innovations adapted to options and barriers.

Innovations can be identified by responding to the characteristics of the households in each segment. Segmentation has been carried out in previous steps in order to adjust innovations to the main characteristics and demands of each segment and the households in each segment should be viewed as customers of the innovation. Different segments require different innovation bundles, adjusted to the respective proximate and underlying barriers. Innovations can then be understood as products which need to match the demands of the customers. Therefore, innovations need to be cost effective, have immediate and long-lasting impacts, and show a high likelihood of substantial productivity gains.

Also, adoption of innovation is not necessarily a binary decision. Rather, the intensity of adoption may change over time, e.g. as a result of learning or through better access to farm resources. The extent of agricultural technology innovation adoption can be measured by intensity of cultivation e.g. cultivated area under the innovation. Then some econometric models, for example, Heckman's two step selection model and Cragg double hurdle model, may be used for estimating the extent of innovation adoption.

The likely productivity growth and livelihood improvement in the different countries (giving priority on marginal areas) for each segment of the poor SHs may be assessed e.g. by modeling or expert consultations. The related literature suggests that the selected methods could be an economic surplus model approach, minimum data, cost/benefit analysis, parametric modeling/graphical, economic modeling, partial budget, or bio-economic modeling approach. Each method has some strength/weakness, data requirements, advantages and disadvantages that need to be carefully reviewed and adjusted to location specific technology innovation prior to using for estimating likely impacts of the innovation on productivity and livelihoods.

Eventually the identified opportunities can be implemented by means of a business plan with prospects of being realized in each partnering country. The business plan discusses how the selected (most promising) agricultural technology innovations identified at the earlier part of the assessment could be implemented.

The business plan needs to elaborate on the requirements and prospective outcomes of adopting particular technology innovations. There should be minimum consent that the adoption of selected innovations is in the interest of the farmers and that it has potential to increase productivity. Among others, the business plan will provide answers to the questions: 1) Where is the SHs' business now (in terms of productivity, livelihood indicators)? 2) What is the objective of an improved business plan? 3) How can that goal be achieved? The business plan can comprise of marketing plan, production plan, management plan, financial plan, and the implementation plan.

For that purpose, relevant stakeholders which have an interest in promoting SHs' productivity (typically those within the value chain but not yet linked) might be brought together in Technology and Business Promotion (TBP) workshops. Alternatively, a business consultant may interview the relevant actor, agree on a set of technologies they would focus on and make informed estimates about the number of farmers reached, the technology applied, the outcomes and the output of the technology. Stakeholders/actors are e.g., farmers, technology providers and producers, credit providers, knowledge providers, input providers (agro-dealers), collective action facilitators (mediators), processors, and wholesalers/purchasers. The business consultant jointly with project partners/local collaborators may select representatives of each stakeholder group for a TBP workshop/interview [29]. A particular case of business plan can be referred at [33].

## 4 Conclusion

The step by step ex-ante assessment we have proposed, states that identifying areas with unused potentials are selected from marginality hotspots (following mapping approach). Unused potentials in those areas have a chance to enhance productivity after introducing agricultural technology innovations. An area and people approach is necessary to capture those unused potentials. In the second step of the assessment, local knowledge for identifying the study villages is gathered and an appropriate study sample is drawn for detailed in-depth investigations. Under the sustainable livelihood framework, stratification is done based on both income poverty measures/social stratification and validated by self-reported perceptions. Then, households from different strata are segmented and cluster analysis is used for this purpose. To see whether underlying barriers affect choice of technology and institutional innovations on the barriers the poor SHs report are supported by the qualitative assessment. In the final step of the assessment, the likely impact of agricultural technology innovations in each segment are assessed by various methods or expert consultation and eventually a business plan for agricultural technology promotion and implementation is prepared.

## Footnotes

1 For, example, today the average farmer in Sub-Saharan Africa gets just over a ton of cereal per acre, while the average Indian farmer gets about twice that, the average Chinese farmer about five times that, and the average American farmer about seven times that amount [6].

2 See[45], [47] for review about poverty, productivity and production environment relationship in the less favored/marginal/laggard areas.

3 Institutions are understood as different forms of rules at different levels of decision making (e.g. norms, conventions, laws, regulations, rights) which are put into play by governance structure, e.g. the market, the state, or particular arrangements to manage the land, like e.g. sharecropping.

## References

[1] Acemoglu D., Robinson J. (2013). Why Nations Fail: the Origins of Power, Prosperity and Poverty.

[2] Acemoglu D. (2009). The crisis of 2008: structural lessons for and from economics. Glob. Growth.

[3] Ahmed A.U., Hill R.V., Smith L.C., Wiesmann D.M., Frankenberger T. (2007). The World's Most Deprived. Characteristics and Causes of Extreme Poverty and Hunger.

[4] Alliance for a Green Revolution in Africa (AGRA) (2012). AGRA's Strategy for an African Green Revolution. http://www.agra-alliance.org/who-we-are/-strategy%5ffor-an-african-green-revolution/.

[5] Annan K.A. (2003). Challenge to the world's scientists. Science.

[6] Bill & Mellinda Gates Foundation (2011). Agricultural Development: Strategy Overview. http://www.gatesfoundation.org/agriculturaldevelopment/Documents/agricultural-%20development-%20strategy-overview.pdf.

[7] Carney D. (1998). Sustainable Rural Livelihoods: what Contribution Can We Make?.

[8] Carney J.P., Maser R.S., Olivares H., Davis E.M., Le Beau M., Yates J.R., Petrini J.H. (1998). The hMre11/hRad50 protein complex and Nijmegen breakage syndrome: linkage of double-strand break repair to the cellular DNA damage response. Cell.

[9] Conway G. (1999). The Doubly Green Revolution: Food for All in the Twenty-first Century.

[10] DeSoto H., Nora D. (2000). Fieldwork Dilemmas: Anthropologists in Postsocialist States.

[11] DFID (2000). Halving World Poverty by 2015: Economic Growth, Equity and Security: Strategies for Achieving the International Development Targets.

[12] Evenson R.E., Gollin D. (2003). Crop Variety Improvement and its Effect on Productivity: the Impact of International Agricultural Research.

[13] Fan S., Hazell P. (2000). Should developing countries invest more in less-favoured Areas? An empirical analysis of rural India. Econ. Political Wkly..

[14] Gatzweiler F., von Braun J. (Feb 2016). Technological and Institutional Innovations for Marginalized Smallholders in Agricultural Development.

[15] Gatzweiler F.W., Braun J.V., Gatwelier F.G. (Feb 2016). Institutional and technological innovations In polycentric systems: pathways for escaping marginality. Technological and Institutional Innovations for Sustainable Intensification of Smallholder Agriculture.

[16] Gatzweiler F.W., Baumüller H. (2014). Marginality—a Framework for Analyzing Causal Complexities of Poverty.

[17] Graw V., Gieseke H., Gatzweiler F.W. (2014). Mapping Marginality Hotspots in Ethiopia.

[18] Graw V., Husmann C. (2014). Mapping Marginality Hotspots.

[19] Gupta A. (2012). Innovations for the poor by the poor. Int. J. Technol. Learn. Innovation Dev..

[20] Hagedorn K.O. (2003). AIster: a “no dead ends” OAI service provider. Libr. Hi Tech..

[21] Hagedorn K. (2008). Particular requirements for institutional analysis in nature-relatedsectors. Eur. Rev. Agric. Econ..

[22] Hazel P.B.R. (2009). Asian Green Revolution. http://www.ifpri.org/sites/default/files/publications/ifpridp00911.pdf.

[23] Herdt R., Pingali P., Evenson R. (2010). Handbook of Agricultural Economics.

[24] Johnston B., Mellor J. (1961). The role of agriculture in economic development. Am. Econ. Rev..

[25] Jones M. (June 2005). Key challenges for technology development and agricultural research in Africa. IDS Bull..

[26] Kuyvenhoven A., Ruben R., Pender J. (2004). Development strategies for less-favored areas. Food Policy.

[27] Leonard H.J. (1989). Environment and the Poor: Development Strategies for a Common Agenda.

[28] Lipton M., Longhurst R. (1989). New Seeds and Poor People.

[29] M.A. Malek, F.W. Gatzweiler, Manual for the identification of idle potentials of marginal rural areas and people by means of technological and institutional innovations. ZEF and BRAC. Available at http://www.zef.de/uploads/tx_zefportal/Publications/TIGA_Manual_2016.pdf.

[31] Malek M.A., Hossain M.A., Saha R., Gatzweiler F.W. (June 2013).

[32] Malek M.A., Hoque M.S., Yesmin J., Haque M.L., Braun J.V., Gatwelier F.G. (Feb 2016). More than cereal-based cropping innovations for improving food and livelihood security of poor smallholders in marginal areas of Bangladesh. Technological and Institutional Innovations for Sustainable Intensification of Smallholder Agriculture.

[33] Mohammad I., Malek M.A. (April 2017). Promotion of agriculture technology in marginal rural areas of Bangladesh: an innovative business model approach. Asian J. Innovation Policy.

[34] North D.C., Wallis J.J., Webb S.B., Weingast B.R. (2007). Limited Access Orders in the Developing World: a New Approach to the Problems of Development.

[35] Ostrom (1990). E.Governing the Commons: the Evolution of Institutions for Collective Action.

[36] Ostrom E. (1994). Constituting social capital and collective action. J. Theor. Polit..

[37] Ostrom E. (1996). Crossing the great divide: coproduction, synergy, and development. World Dev..

[38] Pender J. (2007). Agricultural Technology Choices for Poor Farmers in Less-favored Areas of South and East Asia.IFPRI Discussion Paper 00709.Environment and Production Technology Division. http://www.ifpri.org/sites/default/files/publications/ifpridp00709.pdf.

[39] Pingali P. (July 31, 2012). Green Revolution: impacts, limits, and the path ahead. PNAS.

[40] Poole N., Buckley C.P. (2006). Innovation Challenges, Constraints and Opportunities for the Rural Poor. http://www.ifad.org/events/gc/29/panel/e/poole.pdf.

[41] Rajalahti R. (2012). Sourcebook Overview and User Guide. in: Agricultural Innovation Systems. An Investment Sourcebook.

[42] Radjou N., Prabhu J., Ahuja S., Roberts K. (March 2012). Jugaad Innovation: Think Frugal, Be Flexible, Generate Breakthrough Growth.

[43] Ravallion M.A. (2009). Comparative Perspective on Poverty Reduction in Brazil, China and India.

[44] Ricketts M. (2005). Poverty, Institutions and Economics: Hernando De Soto on Property Rights and Economic Development. Econ. Aff..

[45] Renkow M. (2000). Poverty, productivity and production environment: a review of the evidence. Food Policy.

[46] Ruben R., Pender J., Kuyvenhoven A., Ruben, Pender, Kuyvenhoven (2007). Sustainable poverty reduction in less favored areas: Problem, options and strategies. Sustainable poverty reduction in less favored areas.

[47] Ruben R., Pender J., Kuyvenhoven A., Ruben Pender, Kuyvenhoven (2007). Sustainable poverty reduction in less favored areas: problem, options and strategies. Sustainable Poverty Reduction in Less Favored Areas.

[48] The Rockefeller Foundation (2006). Africa's Turn-a New Green Revolution for the 21st Century. http://m.rockfound.org/uploads/files/dc8aefda-bc49-4246-9e92-9026bc0eed04-africas_turn.pdf.

[49] Reardon T., Kevin C., Bart M., Lourdes A. (2012). The quiet revolution in staple food value chains-Enter the Dragon, the Elephant, and the Tiger. ADB IFPRI.

[50] Von Barun J., Gatzweiler F.W. (2014). Marginality—an Overview and Implications for Policy.

[51] World Bank (1990). World Development Report 1990: Poverty. https://openknowledge.worldbank.org/handle/10986/5973.

[52] World Bank (2007). World Development Report 2008.

